# AWRK6, A Synthetic Cationic Peptide Derived from Antimicrobial Peptide Dybowskin-2CDYa, Inhibits Lipopolysaccharide-Induced Inflammatory Response

**DOI:** 10.3390/ijms19020600

**Published:** 2018-02-17

**Authors:** Qiuyu Wang, Lili Jin, Huan Wang, Sijia Tai, Hongsheng Liu, Dianbao Zhang

**Affiliations:** 1 School of Life Science, Liaoning University, Shenyang 110036, China; qiuyuwang@lnu.edu.cn (Q.W.); lilijin@lnu.edu.cn (L.J.); wanghuan.lnu@gmail.com (H.W.); taisijia.lnu@gmail.com (S.T.); 2 Research Center for Computer Simulating and Information Processing of Bio-macromolecules of Liaoning Province, Liaoning University, Shenyang 110036, China; liuhongsheng@lnu.edu.cn; 3 Department of Stem Cells and Regenerative Medicine, Key Laboratory of Cell Biology, Ministry of Public Health of China, and Key Laboratory of Medical Cell Biology, Ministry of Education of China, China Medical University, Shenyang 110122, China

**Keywords:** AWRK6, antimicrobial peptide, lipopolysaccharides (LPS), inflammatory response

## Abstract

Lipopolysaccharides (LPS) are major outer membrane components of Gram-negative bacteria and produce strong inflammatory responses in animals. Most antibiotics have shown little clinical anti-endotoxin activity while some antimicrobial peptides have proved to be effective in blocking LPS. Here, the anti-LPS activity of the synthetic peptide AWRK6, which is derived from antimicrobial peptide dybowskin-2CDYa, has been investigated in vitro and in vivo. The positively charged α-helical AWRK6 was found to be effective in blocking the binding of LBP (LPS binding protein) with LPS in vitro using ELISA. In a murine endotoxemia model, AWRK6 offered satisfactory protection efficiency against endotoxemia death, and the serum levels of LPS, IL-1β, IL-6, and TNF-α were found to be attenuated using ELISA. Further, histopathological analysis suggested that AWRK6 could improve the healing of liver and lung injury in endotoxemia mice. The results of real-time PCR and Western blotting showed that AWRK6 significantly reversed LPS-induced TLR4 overexpression and IκB depression, as well as the enhanced IκB phosphorylation. Additionally, AWRK6 did not produce any significant toxicity in vivo and in vitro. In summary, AWRK6 showed efficacious protection from LPS challenges in vivo and in vitro, by blocking LPS binding to LBP, without obvious toxicity, providing a promising strategy against LPS-induced inflammatory responses.

## 1. Introduction

Lipopolysaccharides (LPS, also known as lipoglycans and endotoxins) are a major component of the outer membrane of almost all Gram-negative bacteria and elicit strong inflammatory responses in animals [1]. The presence of endotoxins in the blood is called endotoxemia [2]. It can induce sepsis, a leading healthcare problem affecting millions of people around the world [3]. Despite improvements in supportive care and the development of potent antimicrobials, the mortality exceeds 20% [4]. Most antibiotics and anti-LPS antibodies have shown little clinical anti-endotoxin activity, such as polymyxin B (PMB), which can neutralize LPS efficiently while side effects restrict its clinical application [5]. Therefore, it is urgent to develop effective strategies for LPS-induced inflammatory responses.

Antimicrobial peptides are produced as a first line of defense against invading pathogens by all multicellular organisms [6,7]. Recent studies have suggested that some antimicrobial peptides, such as LL-37 and β-defensin, have the potential to protect septic mice from death by directly binding with LPS or by blocking LPS binding to LPS-binding protein (LBP) [8,9,10,11]. In our previous studies, from the skin of the Chinese frog *Rana dybowskii*, dybowskin-2CDYa (SAVGRHSRRFGLRKHRKH, GenBank: ACF08009.1) was isolated and characterized as a novel antimicrobial peptide and further modified by replacing Arg with Lys into the sequence of SWVGKHGKKFGLKKHKKH (named AWRK6), which was effective in antimicrobial and resistant against trypsin [12,13], but the availability of AWRK6 on LPS neutralization and LPS-induced inflammatory responses is unknown.

In the present study, we aimed to investigate the inhibition potency of the synthetic cationic peptide AWRK6 in LPS-induced inflammatory responses, as well as its toxicity, in vivo and in vitro.

## 2. Results

### 2.1. AWRK6 Inhibited the Binding of LPS with LBP in Vitro

**Figure 1:**
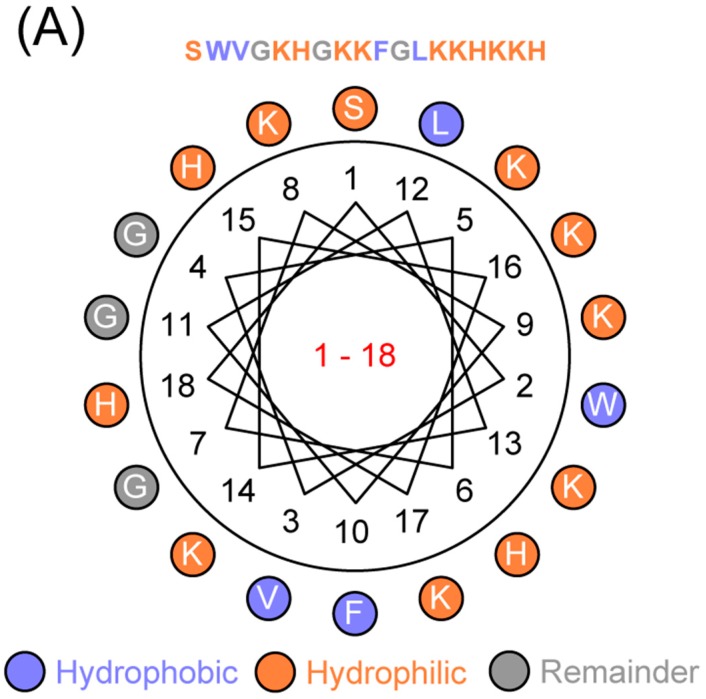

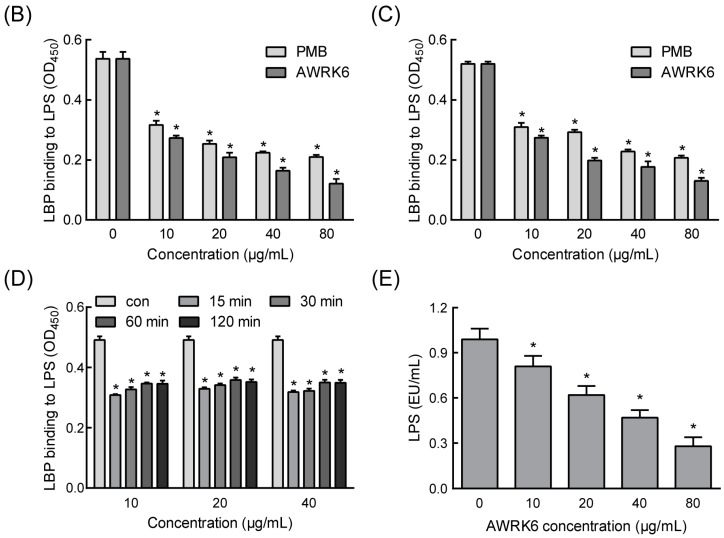
AWRK6 inhibited the binding of lipopolysaccharides (LPS) with LPS binding protein (LBP) in vitro. (**A**) The helical wheel projection of AWRK6. (**B**) The inhibition effects of AWRK6 on the binding of LBP with LPS when AWRK6 was added 1 h before LBP. (**C**) The inhibition effects of AWRK6 on the binding of LBP with LPS when AWRK6 and LBP were added at the same time. (**D**) The inhibition effects of AWRK6 on the binding of LBP with LPS when AWRK6 was added after incubation with LBP for 15, 30, 60, and 120 min. (**E**) The neutralization effects of AWRK6 on LPS, detected by LAL assay. * *p* < 0.05 compared with the 0 (**B**, **C**, and **E**) or con (**D**) groups.

The helical wheel projection showed that the α-helical peptide AWRK6 was positively charged (+11.315) and amphipathic (Figure 1A). The efficacy of the peptide in LPS neutralization was examined using an enzyme-linked immunosorbent assay (ELISA), PMB was used as a positive control. As shown in Figure 1B,C, when AWRK6 was added 1 h before LBP or at the same time, the binding of LBP with LPS was reduced to less than 50% vs. the control in a dose-dependent manner; when AWRK6 was added after incubation with LBP for 15, 30, 60, and 120 min, the binding of LBP with LPS was reduced to about 70% and showed a time-dependent increase (Figure 1D). Furthermore, AWRK6 showed significantly better inhibition effects than PMB on the binding of LBP with LPS at the same concentrations (Figure 1B,C). In addition, to address the issue whether the peptide is binding with LPS or LBP, the LAL assay has been carried out to detect AWRK6-LPS interaction. As shown in Figure 1E, the LPS concentration was significantly decreased by the treatment with AWRK6 in a concentration-dependent manner. Thus, we concluded that the peptide AWRK6 is binding with LPS. These results indicated that AWRK6 can inhibit the initial step of LPS-induced inflammatory response in vitro and may serve as a potential novel therapy strategy.

### 2.2. AWRK6 Protects Mice from Endotoxemia

**Figure 2:**
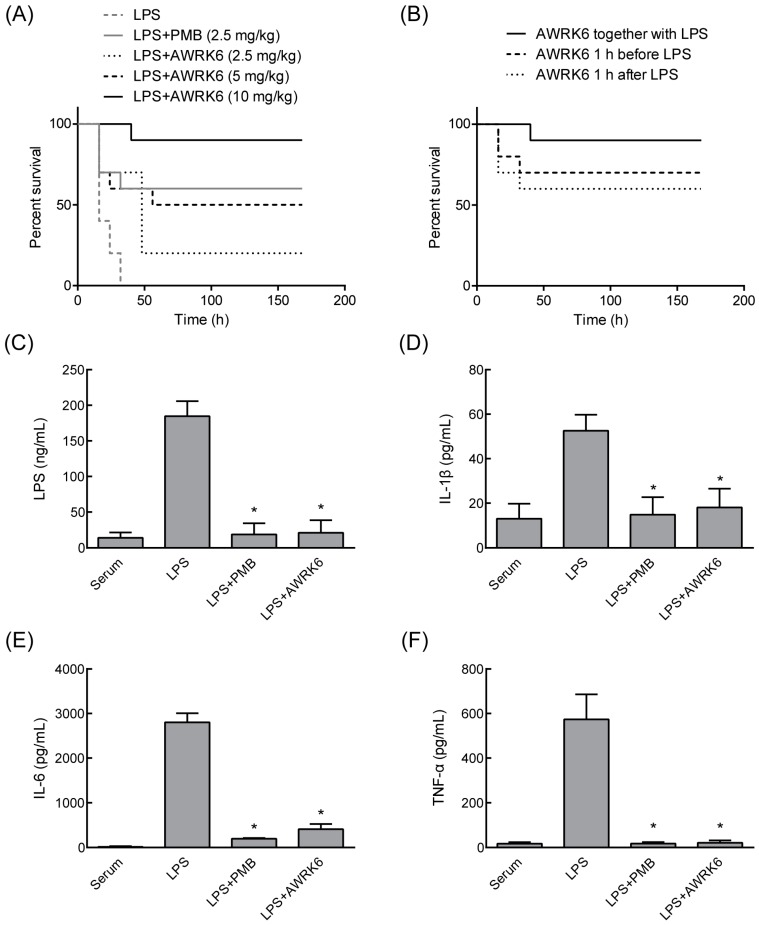
AWRK6 prevented LPS-induced lethal endotoxemia in mice. (**A**) The survival curves of LPS-induced endotoxemic mice treated with a single dose of AWRK6 (2.5, 5, or 10 mg/kg, *n* = 30). (**B**) The survival curves of endotoxemic mice treated with 10 mg/kg AWRK6 at different time points (*n* = 30). (**C**–**F**) The effects of AWRK6 treatment (10 mg/kg) on the serum levels of LPS, IL-1β, IL-6, and TNF-α in the endotoxemic mice. PMB was used as a positive control. * *p* < 0.05 compared with the LPS group.

To evaluate the in vivo activity of AWRK6 against LPS-induced inflammatory response, we constructed a model of murine endotoxemia [14]. As shown in Figure 2A, after the mice were challenged with LPS, the LPS group developed the symptom of endotoxemia, and the mice started to die at 16 h and completed at 32 h. Simultaneous treatment with a single dose of AWRK6 (2.5, 5, or 10 mg/kg) conferred significant protection from lethal endotoxemia within 168 h in a dose-dependent manner vs. the LPS group serving as a control. The survival rates of mice at 168 h were 20%, 50%, and 90% in the groups treated with 2.5, 5, and 10 mg/kg AWRK6 intraperitoneally. The PMB positive control (2.5 mg/kg) offered a 60% protection from the lethal shock. Additionally, pretreatment and delayed treatment with AWRK6 (10 mg/kg) also showed satisfactory protection efficiency against endotoxemia (Figure 2B). The treatment of endotoxemic mice with AWRK6 (10 mg/kg) significantly attenuated the serum levels of LPS, IL-1β, IL-6, and TNF-α (Figure 2C–F), indicating that it prevented endotoxin-induced lethality by attenuating the release of early systemic mediators of lethality.

**Figure 3:**
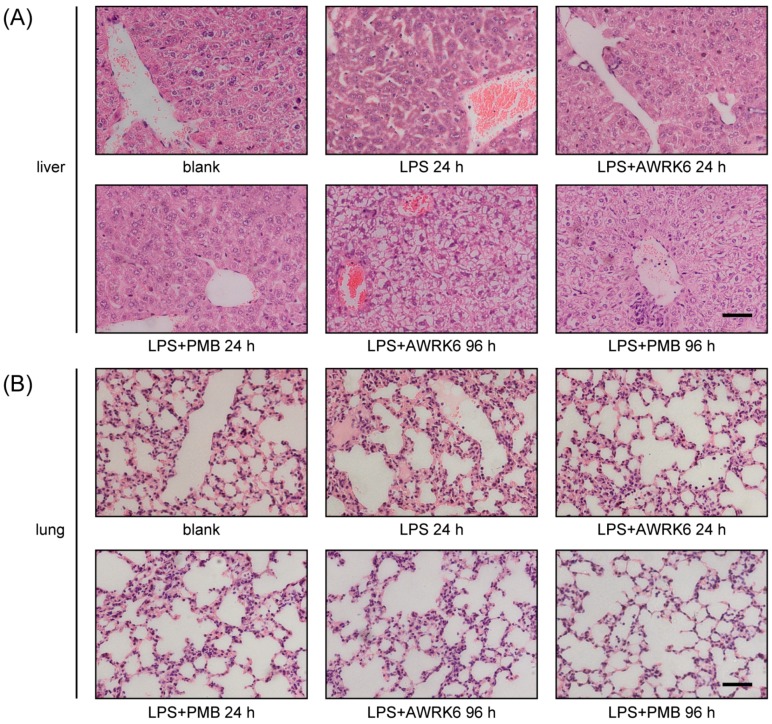
AWRK6 protected liver and lung injury in endotoxemia mice. (**A**) Histopathological analysis of HE-stained sections from liver in endotoxemia mice. (**B**) Histopathological analysis of HE-stained sections from lung in endotoxemia mice. PMB was used as a positive control. Bar indicates 100 nm.

To further investigate the protective effect of AWRK6 on liver and lung injury in endotoxemia mice, histopathological analysis of HE-stained sections from liver and lung was performed. As shown in Figure 3A, normal morphological structures of liver were observed in the blank control, while LPS treatment for 24 h induced acute inflammatory response characterized by decreased cell density, an enlarged cell gap, and neutrophils infiltration. These changes were reversed by the treatment with AWRK6 for 24 h and returned to normal after AWRK6 treatment for 96 h. Meanwhile, LPS treatment resulted in evident inflammatory response in the lungs. As shown in Figure 3B, LPS-exposed mice developed lung surface patchy hemorrhage, inflammatory cell infiltration, interstitial thickening, and the destruction of lung parenchyma. However, after AWRK6 treatment, the pathological changes in the lung tissues were relieved in a time-dependent manner. These results suggested that AWRK6 could improve the healing of liver and lung injury in endotoxemia mice, recovering the physiological status.

### 2.3. AWRK6 Modulated LPS-Activated Pro-Inflammatory Mediators

**Figure 4:**
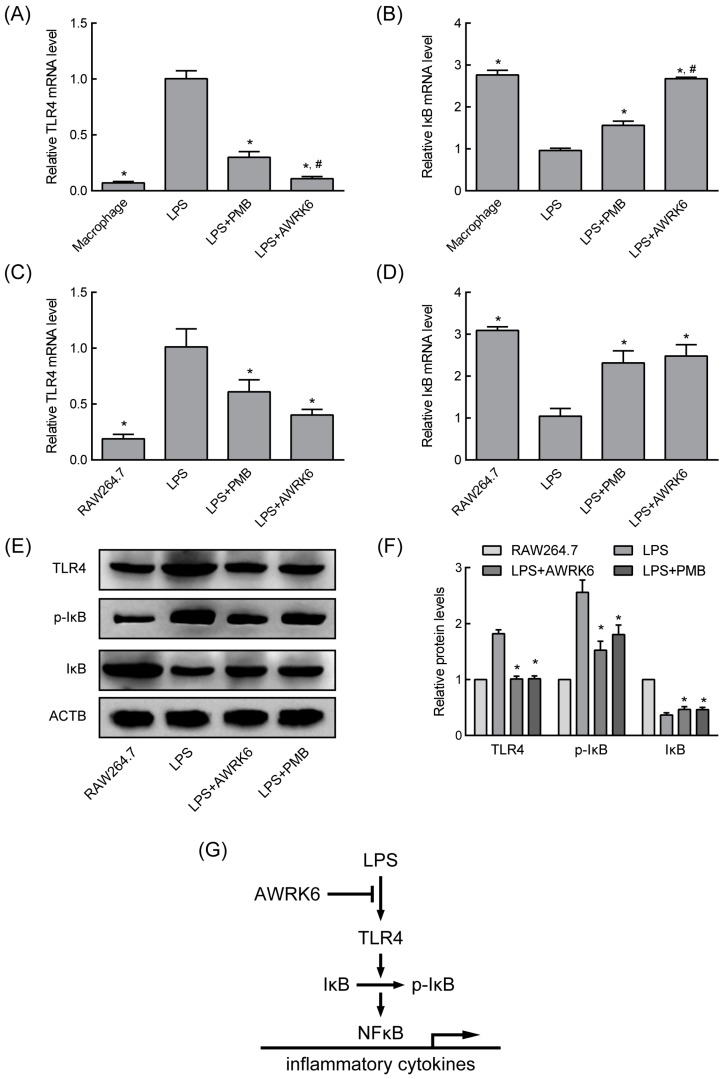
AWRK6 relieved LPS-induced systemic immune response by blocking TLR4/NF-κB activation. (**A**,**B**) The mRNA levels of two genes involved in immune response, TLR4 and IκB in primary macrophages were analyzed by real-time PCR. (**C**,**D**) The mRNA levels of TLR4 and IκB in RAW 264.7 cells were analyzed by real-time PCR. (**E**) The expression of TLR4, IκB, and p-IκB was detected by Western blotting. (**F**) The quantification of Western blotting was applied with ImageJ. (**G**) AWRK6 inhibited LPS-induced immune response by blocking TLR4/NF-κB activation (the arrows indicate induction and T bar indicates inhibition). * *p* < 0.05 compared with LPS group, # *p* < 0.05 compared with the LPS + PMB group.

To gain more insight into the consequences of AWRK6 treatment on LPS-induced inflammatory response, TLR4 and IκB expression in primary macrophages and RAW 264.7 cells were determined by real-time PCR. After the macrophages were challenged with LPS, the innate immunity mediator TLR4 was significantly enhanced (Figure 4A,C) and the NF-κB inhibitor IκB was significantly reduced (Figure 4B,D). When incubated with 20 μg/mL AWRK6 or PMB (positive control), these changes were reversed, indicating the blocking effects of AWRK6 on the LPS-induced TLR4 activation. These results were also confirmed by Western blotting in protein level (Figure 4E,F). For the phosphorylation is critical to IκB functions, the phosphorylation of IκB was further examined. As shown in Figure 4E,F, LPS-induced p-IκB reduction was revised by AWRK6 or PMB treatment. These data suggest that AWRK6 could remedy LPS-induced systemic immune response by blocking TLR4/NF-κB activation (Figure 4G).

### 2.4. The Toxicity of AWRK6 in Vivo and in Vitro

**Figure 5:**
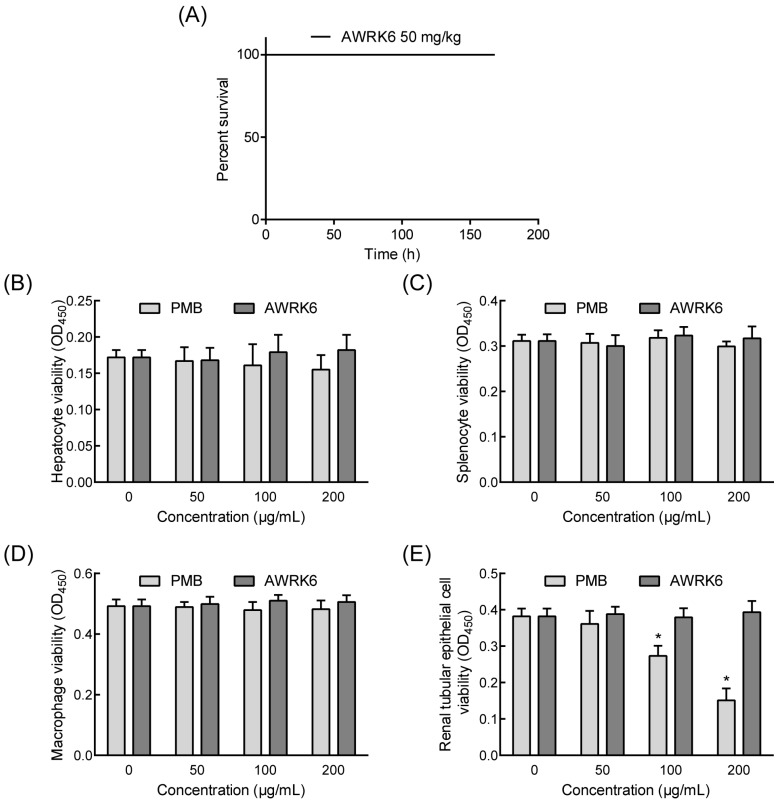
AWRK6 did not produce any significant toxicity in vivo and in vitro. (**A**) The survival curves of mice treated with a single dose of AWRK6 (*n* = 10). (**B**–**E**) The cell viabilities of primary murine hepatocytes, splenocytes, macrophages, and renal tubular epithelial cells incubated with AWRK6 and PMB. * *p* < 0.05 compared with the 0 group.

AWRK6 showed great efficiency in LPS neutralization, it has a potential application in anti-LPS treatment. Therefore, it is important to determine the safety of AWRK6 in vivo and in vitro. In mice, a single dose of AWRK6 (100 mg/kg) administrated through intraperitoneal injection induced no death for 168 h (Figure 5A). Moreover, primary murine hepatocytes, splenocytes macrophages, and renal tubular epithelial cells were isolated and treated with AWRK6 or PMB at 50–200 μg/mL for 24 h. There was no significant difference in cell viability for AWRK6-treated groups, while 100 and 200 μg/mL PMB reduced the cell viabilities of the renal tubular epithelial cells (Figure 5B–E). Under the conditions of these tests, AWRK6 did not produce any significant toxicity in vivo and in vitro.

## 3. Discussion

The treatment of LPS-induced inflammatory responses mainly relies on anti-infection therapies and supportive care [15]. The application of a large amount of broad-spectrum antibiotics produces many side effects such as liver and kidney toxicity and multidrug-resistant bacteria [16]. More seriously, a high amount of endotoxin is released when antibiotics inhibit and kill Gram-negative bacteria, and there are few clinically effective cures that inhibit LPS-activated inflammatory responses [15,17]. Great efforts have been made on the development of anti-LPS agents, such as peptides or anti-LPS antibodies for LPS neutralization, LPS antagonists for competitive combination with LBP, and inhibitors for relative pathways [18,19]. Recently, several antimicrobial peptides have been reported to inhibit LPS-induced pro-inflammatory response to protect septic mice by binding with LPS, indicating a promising approach to fight LPS [8,10]. Considering that LPS-induced inflammatory responses are involved in complex pathways and inflammatory mediators, it is unlikely for the blockage of single downstream targets to relieve the overall outcome [20,21]. Thus, LPS neutralizing agents, such as antimicrobial peptides and PMB, which prevent the initiation of the inflammatory responses, may have better value. In the present study, we found that the novel synthetic cationic peptide AWRK6 could neutralize LPS to inhibit LPS-induced inflammatory responses in vivo and in vitro, without obvious toxicity.

The anti-LPS activity of antimicrobial peptides depends on their structural and biochemical properties [22]. In this study, AWRK6 was an α-helical peptide with a high net positive charge, which is beneficial to binding with negatively charged LPS. In terms of the LPS neutralizing effect of AWRK6, when AWRK6 was added before LBP or at the same time, the neutralization efficiency was greater than 50%, which could be regarded as a precaution against an LPS-induced inflammatory reaction. When AWRK6 was added after LBP, the neutralization efficiency was about 30%; this could be considered the therapeutic effect, which is more important. LPS in the blood could induce the expression of a variety of inflammatory factors and result in multiple organ injury in, for example, the liver and lung, leading to the death of shock [23]. In the endotoxemic mice model, via AWRK6 treatment, LPS and inflammatory cytokines in the serum were significantly reduced, the liver and lung injuries were reversed, and the LPS-induced lethal endotoxemia was effectively cured. In addition, there was no significant toxicity of AWRK6 observed in primary murine hepatocytes, splenocytes, macrophages, renal tubular epithelial cells, or mice. These data show the impressive endotoxin-neutralization activity of AWRK6 in vivo and in vitro and that it has the potential to become a novel anti-endotoxin agent.

LPS-induced inflammatory responses are mainly activated by the interaction of LPS-LBP-CD14 complexes with TLR4 to send signals into the cells [24]. The activation of TLR4 could promote IκB phosphorylation and degradation, stimulating NF-κB activation, which induces early proinflammatory factors such as IL-1β, IL-6, and TNF-α release [25,26]. Recent studies indicated that LL-37 and β-defensin could suppress LPS-induced cellular cytokines release by binding directly to LPS or by blocking LPS binding to LBP [8,10]. In this study, AWRK6 significantly revised LPS-induced TLR4 overexpression and IκB depression, as well as enhanced IκB phosphorylation. Thus, it was in the initial stage that AWRK6 inhibited LPS-induced inflammatory responses, by blocking the activation of TLR4/NF-κB pathway.

In summary, AWRK6 showed efficacious protection from LPS challenge in vivo and in vitro, by blocking LPS binding to LBP, without obvious toxicity, thus offering a promising novel strategy against LPS-induced inflammatory responses. 

## 4. Materials and Methods

### 4.1. Sequence Analyses

The primary sequence analysis of AWRK6 was performed using ANTHEPROT 6.9.1, and the secondary structure was predicted by the Grbrat method [27].

### 4.2. Peptide Synthesis

The peptide AWRK6 were synthesized by GL Biochem Corporation (Shanghai, China) using solid-phase synthesis method, and the molecular weights were confirmed by matrix-assisted laser desorption/ionization time of flight mass spectroscopy (MALDI-TOF MS, Autoflex, Bruker Daltonics, Bremen, Germany). Subsequently, the peptide was purified by reverse-phase high-performance liquid chromatography (RP-HPLC) and salt conversion was applied for TFA removal, for a high purity of more than 99%. The lyophilized peptide was dissolved in physiological saline for the in vivo and in vitro experiments.

### 4.3. ELISA

The blockade effects of AWRK6 or PMB on the LBP binding to LPS were evaluated by sandwich ELISA. In short, 96-well ELISA plates (NUNC MaxiSorp, Shanghai, China) were coated with 100 μL of LPS (100 ng/mL, Sigma, Shanghai, China) overnight at 4 °C and blocked with 10% BSA at 37 °C for 1 h. AWRK6 or PMB (Wako, Kanagawa, Japan) were added with LBP (CLOUD-CLONE, Wuhan, China) at the indicated time. AWRK6 or PMB was added 1 h before LBP, at the same time and at 15, 30, 60, or 120 min after LBP. The mouse anti-LBP monoclonal antibody (1:1000, Santa Cruz, Dallas, TX, USA) was added with/without AWRK6 or PMB and incubated at 37 °C for 1 h, followed by incubation with peroxidase-conjugated goat anti-mouse IgG (1:1000, Santa Cruz) for an additional 1 h at room temperature. TMB (Solarbio, Beijing, China) was applied for the detection using a microplate reader iMARK (Bio-Rad, Hercules, CA, USA) at 450 nm.

For the detection of LPS, IL-1β, IL-6, and TNF-α in eyeball serum, the serum was collected 3 h after treatment of 10 mg/kg AWRK6 in the mouse endotoxemia models. Commercial ELISA kits (LPS kit from CLOUD-CLONE, Wuhan, China; IL-1β, IL-6, and TNF-α kits from MultiSciences, Hangzhou, China) were applied according to the manufacturer’s instructions.

### 4.4. LAL Assay

LAL assay has been carried out to detect AWRK6-LPS interaction using ToxinSensor Chromogenic LAL Endotoxin Assay Kit (GenScript, Nanjing, China). According to the manufacturer’s instructions, LPS were incubated with 0, 10, 20, 40, and 80 μg/mL AWRK6 at 37 °C for 15 min; 100 μL of reconstituted LAL were added and mixed well. The mixture was incubated at 37 °C for 45 min and mixed with 100 μL of reconstituted chromogenic substrate solution. After incubation at 37 °C for 6 min, 500 μL of Stop Solution was added. The absorbance at 570 nm was detected using a microplate reader iMARK.

### 4.5. Mouse Endotoxemia Models

Female Kunming mice (18–22 g) were provided by the Experimental Animal Research Center of Shenyang Medical College (Shenyang, China). Mice were housed in individual cages with free access to food and water under controlled temperature (18–22 °C). All mice were acclimated to housing conditions for at least 5 days. The animal experiments were approved by the Ethics Committee of Liaoning University (20150011, 23 February 2015) and conducted according to the ethical guidelines for the Care and Use of Laboratory Animals.

To develop mouse endotoxemia models, LPS (50 or 100 mg/kg) were administrated through intraperitoneal injection. AWRK6 or PMB was also intraperitoneally administered in sterile saline at the indicated concentrations (2.5, 5, and 10 mg/kg) and time points (AWRK6 or PMB added 1 h before LBP, at the same time, and 1 h after LBP). An equal volume of sterile saline was used as a control. The mortality rates were recorded every 8 h for 168 h after LPS injection.

### 4.6. Histopathological Examination

For histopathological evaluation, the livers and lungs were harvested and fixed in 10% formalin for at least 24 h, followed by paraffin embedding. Then, the tissue sections (5 μm) were made and stained with hematoxylin and eosin (Solarbio) using standard procedures. Histopathology analyses were carried out under light microscopy (Olympus, Tokyo, Japan).

### 4.7. Cell Isolation and Culture

The murine macrophage cell line RAW 264.7 was purchased from CHI Scientific (Shanghai, China). The cells were maintained in Dulbecco’s Modified Eagles Medium (DMEM) (HyClone, Beijing, China) supplemented with 10% fetal bovine serum (CellMax, Beijing, China) and 1% Penicillin streptomycin (Solarbio) at 37 °C in a humidified atmosphere containing 5% CO_2_. For experiments, the cells were seeded in 6-well plates and treated with LPS (100 ng/mL), meanwhile AWRK6 (20 μg/mL) or PMB (20 μg/mL) were added as an antagonist. Total RNA and total protein were prepared after 2 h of incubation.

The peritoneal macrophages, liver cells, spleen lymphocytes, and renal tubular epithelial cells from mice were isolated using general protocols [28]. Briefly, the mice were euthanized by rapid cervical dislocation. For macrophages, 5 mL of ice-cold PBS were injected into the abdomen and after a massage for 3 min, the fluid was withdrawn and centrifuged to collect macrophages. For liver cells, the livers were washed with ice-cold PBS and treated with collagenase type II (Solarbio) for 1 h, followed by filtering and centrifugation. For spleen lymphocytes, spleen tissues were collected. Splenocytes were prepared by disrupting the spleen with a grinder, and erythrocytes were lysed using Red Blood Cell Lysis Buffer after centrifugation. For renal tubular epithelial cells, the kidney tissue was sheared, ground, washed twice with PBS, and then incubated with 1 mg/mL collagenase I for 20 min. The cells were washed with PBS before seeding. The macrophages, liver cells, and spleen lymphocytes were cultured in 1640 medium (HyClone) supplemented with 10% fetal bovine serum (HyClone) and 1% penicillin streptomycin (Solarbio). The renal tubular epithelial cells were cultured in DMEM/F12 (HyClone) medium supplemented with 10% fetal bovine serum (HyClone) and 1% penicillin streptomycin (Solarbio).

### 4.8. Quantitative Real-Time PCR

Total RNA was isolated using TRIzon Reagent (CW biotech, Beijing, China) according to the manufacturer’s instructions. Reverse transcription was carried out using FastKing-RT SuperMix (Tiangen, Beijing, China) and quantitative real-time PCR was performed using UltraSYBR Mixture (CW biotech) in ABI 7500 Real-Time PCR System. The relative gene expression was calculated using the ΔΔ*C*_t_ method, and ACTB was used as an internal control. Primers were synthesized by Sangon Biotech (Shanghai, China) and the sequences are given in Table 1.

### 4.9. Western Blotting

Total protein was prepared using RIPA lysis buffer (Beyotime, Haimen, China) and quantified by BCA protein assay kit (Beyotime). Equal amounts of proteins were separated by 10% SDS-PAGE, trans-blotted onto an NC membrane, and incubated with rabbit anti-IκB α antibody (1:2000, Abcam, Cambridge, MA, USA), rabbit anti-IκB α antibody (phospho S36, 1:5000, Abcam), rabbit anti-TLR4 antibody (1:2000, Abcam), and mouse anti-β actin mAb (1:5000, ZSGB BIO, Beijing, China) at 4 °C overnight. After probing with HRP-conjugated secondary antibodies (Dingguo, Beijing, China) at room temperature for 1 h, protein bands were visualized using ECL Plus Reagent (Solarbio) on DNR chemiluminescence detection system. The bands were analyzed using ImageJ software.

### 4.10. Cytotoxicity Analysis

A CCK-8 assay (CCK-8, Dojindo, Kumamoto, Japan) was used to investigate the cytotoxicity of AWRK6 in murine peritoneal macrophages, liver cells, spleen lymphocytes, and renal tubular epithelial cells. Cells were seeded in 96-well plates and treated with AWRK6 or PMB at 5, 10, and 20 μg/mL for 24 h. After 10 μL of CCK-8 reagent were added to each well, the cells were continuously incubated for 2 h. The spectrophotometric absorbance was measured using an iMARK microplate reader (Bio-Rad).

### 4.11. Statistical Analysis

The survival rates were compared between groups by the Mantel–Cox Log-rank test. Other experiments were performed in triplicate and statistical analyses were performed using a Student’s *t*-test or a one-way analysis of variance (ANOVA). *p* < 0.05 Was considered statistically significant. All statistical analyses were performed using GraphPad Prism 6 software (GraphPad Software, Inc., La Jolla, CA, USA).

## Figures and Tables

**Table 1:** Primer sequences for real-time PCR.

Gene	Primer Sequence
*TLR4*	Forward: 5′-TCTGCCTTCACTACAGAGACT-3′
	Reverse: 5′-AGTCTTCTCCAGAAGATGTGC-3′
*JNK*	Forward: 5′-TATACGCATAAGTACGGCTACA-3′
	Reverse: 5′-GTCCTGGTGGGAAATGAAC-3′
*IκB*	Forward: 5′-GCCCTTCTGGGATTTCCT-3′
	Reverse: 5′-GCGGCTCCGCTTCGTTCT-3′
*ACTB*	Forward: 5′-TTGTTACCACCTGGGACG-3′
	Reverse: 5′-GGCATAGAGCTCTTTACGG-3′
